# Supplementary material for: Are carbon emissions trading and green financial instruments synergistic? -Comprehensive quantitative research based on content analysis
Source: PLoS One. 2024 Mar 7;19(3):e0298601. doi: 10.1371/journal.pone.0298601 (PMC10919601; doi:10.1371/journal.pone.0298601)
Supplement: S1 Data — (ZIP) [file pone.0298601.s001.zip › Data/地级市Z_F工作报告.zip!/─┌├╔╣┼/║⌠┬╫▒┤╢√╩╨/└┤╫╘╣1⁄2╓┌║┼í╛┬φ┐╦╩2╛▌═°í┐_2015.docx]

各位代表：

现在，我代表市人民政府向大会报告工作，请予审议，并请市政协委员和列席会议的同志提出意见。

一、2014年工作回顾

过去的一年，是我市发展进程中不平凡的一年。面对市场需求不足、宏观经济下行压力持续加大等复杂严峻形势和艰巨繁重的改革发展稳定任务，在自治区党委、政府和市委的正确领导下，市政府团结带领全市各族人民，深入贯彻自治区“8337”发展思路和市委三届五次全委会议暨经济工作会议精神，统筹保增长、调结构、促改革、惠民生，经济社会呈现出总体平稳、稳中有进的发展态势，开创了建设现代化呼伦贝尔的新局面。

（一）经济保持平稳增长。地区生产总值完成1522.3亿元,增长8.4%。公共财政预算收入完成96亿元，增长10.2%。500万元以上项目固定资产投资完成803亿元①，增长16%。神华褐煤综合利用、圣山褐煤清洁高效利用、扎兰屯支线机场、滨洲铁路电气化改造、大兴安岭北部原始森林国家公园、尼尔基水库下游30万亩灌区、Z866水利枢纽等11个重大项目获得国家核准和出具路条，总投资近700亿元，将有力支撑产业转型发展。社会消费品零售总额完成501.6亿元，增长11.5%。城镇常住居民人均可支配收入和农村牧区常住居民人均可支配收入分别完成24787元和10751元，增长9.6%和11.5%，继续高于经济增速。完成了自治区下达的节能节水减排等约束性指标任务。

（二）结构调整取得新进展。三次产业结构演进为18.1:46.8:35.1。农牧业结构调整成效显著。牧业年度牲畜存栏1955.4万头只, 畜牧业产值占第一产业比重达34%;农区牲畜头数达1421.5万头只，占全市牲畜总数的72.7%。粮食产量123亿斤②，玉米种植面积超过1000万亩，取得重大突破。销售收入500万元以上农牧业产业化龙头企业销售收入增长10.7%，达到397亿元，占规模以上工业产值的31%。

工业内部结构不断优化。农畜产品加工、生物制药等非资源型产业实现产值865亿元，占工业总产值的67.3%，增长15.3%，高于资源型产业增速12.4个百分点。加快推进资源型产业延伸升级，全年发电量340亿度,煤化工形成了110万吨化肥、20万吨甲醇生产能力,有色金属形成了日采选矿石9万吨和年冶炼20万吨生产能力。

现代服务业繁荣发展。旅游人数和收入达1293.5万人次、364.2亿元，分别增长13.4%和30.6%。呼伦贝尔万家惠农贸批发零售市场等32个重点商贸物流项目完成投资37亿元，批发零售销售额和住宿餐饮营业额增速仍保持较快增长。

非公经济增加值占全市地区生产总值52%，拉动就业2.6万人，占全市新增就业人数的76%。

（三）发展活力和动力明显增强。认真落实自治区部署的改革任务，年度改革事项扎实推进。政府机构改革如期完成，食品药品监管职能整合、工商质监体制调整理顺到位。市本级行政许可事项精简比例达51.3%，保留的审批事项完成流程再造。政务服务中心和公共资源交易中心启动运行，39个部门和单位入驻政务服务中心，政府采购、工程建设招投标、土地使用权、矿业权和农村牧区土地、草牧场、林权等交易进入公共资源交易中心。公布56类283项政府购买服务事项，政府购买服务正式推开。

商事制度改革成效显著。在全区率先实行了“先照后证”改革和注册资本认缴制，全市新注册企业3056户，增长127%；新登记市场主体注册资本达181.4亿元，增长191.3%，对促进创业增加就业发挥了关键性作用。新组建了呼伦贝尔旅游、旅业和林业集团，大型投融资建设主体达到9个。市场直接融资取得零的突破，有三家中小企业分别在上海股权交易中心、天津股权交易所挂牌。人民币各项存款余额1156.5亿元，增长4.1%，贷款余额733.2亿元，增长18.5%，社会融资总规模达1305.4亿元，增长17.5%，存贷比和社会融资总量均实现了历史性突破，保障了投资的持续增长。农村牧区改革持续推进。阿荣旗农村土地确权登记颁证和新右旗草原确权承包试点稳步推开，全市流转土地953万亩、草牧场1590万亩。

（四）发展条件不断改善。编制完成了呼伦贝尔市新型城镇化发展规划和城乡体系规划。城市建设完成投资385.2亿元，房屋开工面积1607万平方米，实施了358个市政公用基础设施项目，城市功能日趋完善。

基础设施建设全面提速。综合交通完成投资98亿元，创历史新高。根河—拉布达林等11个重点公路在建项目顺利推进，其中金边壕—大兴等3条公路通车。阿荣旗—莫旗等重点铁路项目进展顺利。扎兰屯支线机场和新右旗等5个通用机场在建项目继续推进。水利完成投资11亿元，扬旗山水利枢纽工程开始蓄水，尼尔基水库下游30万亩灌区和扎敦水利枢纽工程开工建设。电网完成投资20亿元，实施项目112个。

生态环境保护与建设持续加强。全面推进呼伦湖生态环境综合治理，实施了“河湖连通”等工程，水面提升1.15米，水域面积达2100平方公里。落实草原保护补助奖励资金5亿元，禁牧2850万亩，草畜平衡7508万亩。治理沙区100万亩。绿色矿山、和谐矿区建设持续推进。造林绿化120万亩，其中重点区域绿化16万亩。开展环保整治“百日行动”，解决环境隐患和重大问题28个，关停违规企业2家。

（五）对外开放水平进一步提升。满洲里国家重点开发开放试验区建设取得新进展，综合保税区已申报国家审批。中俄蒙合作先导区建设规划和实施方案上报国家。中蒙跨境经济合作区建设纳入中蒙两国元首会谈备忘录和中蒙中长期合作纲要。蒙古国驻呼伦贝尔领事馆正式开展业务。“中俄”、“中欧”货运专列常态化运行。全年引进国内（市外）资金841.5亿元。外贸进出口总额完成36.6亿美元，增长54.1%，实现了逆势增长。

（六）保障和改善民生力度持续加大。公共财政继续向民生倾斜，支出215亿元，占支出总额的61.5%。创业就业工程取得实效，城镇新增就业3.4万人，高校毕业生就业9820人，农牧民转移就业8.2万人，城镇登记失业率控制在3.8%以内。扶贫攻坚工程扎实开展，实施了金融扶贫、金种子扶贫工程，投资10.6亿元，脱贫3.3万人，减贫幅度达25.5%。“十个全覆盖”工程全面启动，投资15.6亿元，424个嘎查村实现了全覆盖，完成总任务量的35%。棚户区改造步伐加快，投资52亿元，地方、森工等各类保障性安居工程开工65414套，开工率102%，建成35492套，完成率115%，开工量和建成量均补齐了以前年度的欠账。社会保障水平稳步提高，城镇退休职工养老金和农村最低生活保障标准提高10%以上，城镇职工“五险”和城乡居民养老保险参保人数均超额完成任务。加大了对困难群众的帮扶力度，发放购煤补助1.4亿元，23.1万低保家庭受益。发放助学金8438万元，帮助2400名低保家庭的孩子圆了大学梦。为每个零就业家庭至少解决1人就业，实现了动态清零。菜篮子工程稳步实施，蔬菜种植面积继续扩大，蔬菜仓储设施达6万立方米,建成63家蔬菜平价超市。居民消费价格指数较上年回落2.4个百分点，降至1.9%，为近5年最低点。中心城区“拆炉并网”工程全面展开，拆除锅炉224台，新增集中供热面积175万平方米。海拉尔北山供水站投入运行，日增供水能力3000吨。

基本公共服务水平全面提高。建设新型研发机构，组建了呼伦贝尔生态产业技术研究院。各级各类教育协调发展，新右旗通过了义务教育发展基本均衡县国家级认定，海拉尔二中分校区项目开工建设。城乡医疗和公共卫生服务体系不断完善，莫旗、根河等四个旗市开展了公立医院改革试点。落实了单独两孩政策，人口自然增长率控制在5‰以下。实施了蒙古族源与元朝帝陵综合研究项目，遗址保护规划编制和文物修复整理有序开展。校园足球事业稳步推进，全民健身和竞技体育蓬勃开展。团委、妇联换届圆满完成。民族宗教、人事人才、档案史志、气象、人防、地震、工会、红十字、关工委、残联和老龄等工作都取得了新进展。

（七）民主法治建设不断加强。认真执行市人大及其常委会决定，自觉接受政协民主监督，广泛听取各民主党派、工商联和无党派人士意见，全年办理人大代表议案1件、建议136件、政协委员提案251件。加强社会主义核心价值观宣传教育，5人荣获“自治区道德模范”称号。扩大农村牧区社区试点范围，全市90%以上的社区办公活动场所达到300平方米以上。清理规范市本级社会组织210个。坚持开展领导干部接访工作，信访总量下降20.9%。加强了安全生产和食品药品安全监管，安全事故起数和死亡人数实现双下降。推进社会治安综合治理，开展平安呼伦贝尔建设工程，双拥和边防工作得到加强。全市民族团结、社会稳定、边疆安宁的大好局面进一步巩固。

（八）政府自身建设取得实效。扎实开展党的群众路线教育实践活动，严格落实中央八项规定、自治区28项配套规定和市委相关要求，着力解决“四风”及联系服务群众“最后一公里”问题。三公经费下降24.5%，清理腾退超标办公用房20.2万平方米，拍卖超标轿车94辆，封停超配越野车239辆。积极推进政务公开，行政监察、审计监督和社会监督得到加强。建立了政府重大行政决策合法性审查制度和法律顾问制度，完善了规范性文件的制定、备案、审查机制。扎实推进了“万名干部下基层大引领行动”。

各位代表！过去一年的成绩来之不易，这是自治区党委、政府和市委正确领导的结果，是全市各族人民守望相助、团结奋斗的结果，是市人大、市政协和社会各界关心、支持、帮助的结果。在此，我代表市人民政府，向全市各族人民，向人大代表、政协委员和各民主党派、人民团体，向驻呼军警部队指战员和所有关心支持呼伦贝尔发展的同志们、朋友们，表示衷心的感谢!

在看到成绩的同时，我们也清醒地认识到我市经济社会发展中还存在一些矛盾和问题，政府工作还有差距和不足。一是市场需求不足、经济下行仍在持续，我们面临的形势复杂严峻，保增长的任务更加艰巨繁重。二是经济结构不优，发展方式粗放，可持续发展能力不强，调结构转方式非常紧迫。三是生态环境、主体功能区、节能减排约束加剧，项目落地困难，投资增长压力加大。四是产能过剩、企业库存增加、销售下滑、利润空间收窄，部分企业亏损严重，生产经营十分困难。五是基础设施建设滞后，生态环境保护与建设需要持续加强。六是主体税种收入下降，新的增长点培育尚需时间，民生支出刚性增长，财政收支矛盾加剧。七是社会建设创新不够，市场机制亟待完善，发展环境需要进一步优化。八是“四风”问题仍不同程度地存在，个别干部为官不为，不作为、慢作为、懒作为现象值得警惕。九是政府职能转变还在路上，法治政府建设任重而道远。对此，我们要高度重视并采取有效措施切实加以解决。

二、2015年主要工作

2015年，是全面深化改革的关键之年，是全面推进依法治国的开局之年，也是全面完成“十二五”规划的收官之年，做好今年的工作意义重大。

今年政府工作的总体要求：以党的十八大，十八届三中、四中全会、习近平总书记系列重要讲话和考察内蒙古重要讲话精神为指导，全面贯彻落实自治区党委九届十一次、十二次、十三次全委会议和市委三届六次全委会暨经济工作会议精神，坚持稳中求进工作总基调，以提高经济发展质量和效益为中心，主动适应经济发展新常态，更加注重投资和项目拉动，更加注重调结构转方式，更加注重改革创新，更加注重向北开放，更加注重建设生态文明，更加注重改善民生，加快建设“五大基地”、“两个屏障”、“一个桥头堡”，加快建设法治政府，坚定信心、压实责任、攻坚克难，全面开创呼伦贝尔美丽发展、科学崛起、共享繁荣的新局面。

今年经济社会发展主要预期目标：地区生产总值增长8.5%左右，限额以上固定资产投资增长13.5%，社会消费品零售总额增长11.5%，公共财政预算收入增长6.5%，城镇和农村牧区常住居民人均可支配收入分别增长10%和11%以上。主要约束性指标达到自治区要求。

完成上述目标，重点把握好以下原则和取向：

主动适应新常态。经过三十多年持续高速增长，我国在需求、市场、资源环境约束、劳动力和资源配置等诸多方面都已发生了趋势性变化，进入了增速换挡、结构调整、动力转换的新常态。我市生态环境、主体功能区和节能减排等约束同时发力。这些，倒逼我们必须牢牢把握新常态下经济发展的大逻辑，更加注重发展的质量和效益，做到观念上适应、认识上到位、方法上对路、工作上得力，推动经济社会持续健康发展。

把调结构转方式放到更加重要的地位。经济下行的主要症结在于经济结构不优。我市经济已到了转型发展、深度调整、整体提升的关键时期。为此，我们要把调整经济结构、转变发展方式作为主动适应新常态的根本举措。紧紧围绕建设“五大基地”、“两个屏障”、“一个桥头堡”的要求，以增量促调整、以存量促转型，努力实现传统产业新型化、新兴产业规模化、支柱产业多元化。

向改革创新要动力。当前，传统增长动力减弱、活力不足，必须寻求新动力、培育新增长点。这里的关键是，要搞活市场，靠市场培育新增长点；要抓实创新，靠新理念、新方法培育新产业；要放宽政策，营造大众创业、市场主体创新的良好环境。以改革创新增强发展动力、释放发展活力。

持续保障和改善民生。这是政府一切工作的出发点和落脚点。要带着责任和感情抓民生，把事关广大人民群众切身利益的问题放在心上，抓在手上，落实在行动上。统筹抓好就业、扶贫、上学、就医、住房、供水、供热、供电以及柴米油盐、衣食住行等与老百姓生活息息相关的各项工作。立足于保基本、兜底线、促公平，解决好困难群众的生产生活，多做雪中送炭的事，让广大人民群众共享改革发展的成果。

今年要重点做好十个方面的工作：

（一）确保经济稳定增长

保增长是经济工作的首要任务，也是艰巨任务，核心和关键是保投资、保项目。

保投资。全年计划安排500万元以上项目固定资产投资910亿元。其中，工业投资290亿元、城市建设投资280亿元、交通及电网基础设施建设投资150亿元、农牧林水及其他投资190亿元。

保项目。按照国家产业政策，发挥自身优势、找准投资方向，既要坚持不懈推动打基础、管长远的重大项目，同时要抓好周期短、见效快、增加就业多的中小项目。全市安排亿元以上重点项目210个。其中，工业类50个、城市建设类70个、基础设施类27个、农业类16个、商贸和民生社会事业类47个。

抓存量。加大对煤炭、电力等经营困难企业的服务力度，帮助企业降低成本，开拓市场，渡过难关。全力抓好经济运行的日常调度，做好原料、融资、争取政策支持等生产经营要素的组织和优化，挖掘、释放闲置生产能力，盘活存量。

促增量。对已经具备生产条件的项目，抓紧配套，一企一策解决具体问题，力推项目投产。已完成报批手续的项目，要做好进场前的各种准备，尽早开工。在谈的项目要加快推进前期工作，确保尽早落地，形成投资拉动。

（二）持续推进新型工业化

着力加强基地建设。清洁能源输出基地，继续推进面向华北、山东的电力外送通道前期工作，开工大杨树和牙克石两个2×20万千瓦热电联产项目。电力装机达到900万千瓦以上。现代煤化工基地，争取蒙东伊敏国家级现代煤化工基地获批，加快神华褐煤综合利用项目、东能化工120万吨和盛虹480万吨二甲醚项目前期，力争圣山褐煤清洁高效综合利用项目开工，宏裕煤制化肥项目投产。煤炭就地转化率达到45%以上。有色金属生产加工基地，争取甲乌拉矿区列入国家勘查规划，继续推进大兴安岭中段整装勘查，加快国森矿业二道河铅锌矿采选项目前期，开工中国黄金10万吨铜冶炼项目，推动驰宏矿业20万吨铅锌冶炼项目达产达效。打造有色金属探、采、选、冶、加全产业链条。绿色农畜产品生产加工输出基地，依托农垦集团、阜丰公司和呼伦贝尔肉业集团等骨干企业，推动农畜产品精深加工。全年加工转化玉米300万吨、加工肉牛10万头、肉羊450万只，全市农畜产品加工转化率达到50%。

壮大新兴产业规模。生物制药产业，推动鸿洋药业一期和北方药业二期投产，力争鸿洋药业二期、北方药业三期开工，带动物流及上下游配套产业发展，逐步形成循环产业优势。玉米发酵产业，开工扎兰屯同联100万吨玉米初加工和阜丰二期10万吨苏氨酸、20万吨谷氨酸钠项目，推动玉米发酵产业向高附加值领域延伸。装备制造业，支持华德牧业机械二期扩建工程，推动山东能源枣矿集团机械装备制造基地建设取得新进展。信息产业，重点推进中网科技、浪潮集团云计算等项目。加快传统产业整合升级。木材加工产业，培育木材加工龙头企业，提高产品附加值和市场占有率，推动满洲里木材加工园区整合升级。建材产业，开工鑫昌泰二期日产4000吨水泥熟料项目，继续推进盛伟科技日产5000吨超细粉煤灰项目。乳品产业，推动雀巢乳业优化产品结构。加大市域乳业企业重组整合力度，优化岭东、岭西乳业布局，提高产业集中度。林下产品加工业，支持森工集团和呼伦贝尔林业集团整合林下产品加工业，形成品牌和规模。

推进工业园区建设。整合资源，明确定位，实施差异化发展。完善园区基础设施，发展园区生产性物流，加快园区规划的环评工作。重点推动呼伦贝尔经济技术开发区、岭东工业园区、伊敏工业园区、扎赉诺尔工业园区等重点园区加快发展。

扶持中小企业发展。健全工作机制，制定中小企业投资方向指导目录。建立中小企业融资、担保、创业和服务平台。推进民贸民品企业发展。鼓励大众创业、万众创新，使中小企业快速发展壮大。

（三）加快发展现代农牧业

推进农牧业产业化进程。扶持现有产业化龙头企业，积极引进加工流通方面的大型龙头企业，提升龙头企业带动作用。鼓励引导企业参与规模化、标准化基地建设，突出抓好肉牛、肉羊和乳业加工。提升扎兰屯绿色食品交易会影响力，建设农畜产品绿色食品区域性大市场，提高市场占有率。加强品牌建设，全力打造呼伦贝尔绿色品牌和地理标志商标。加快农畜产品质量安全标准体系和追溯体系建设。

推动农牧业内部结构调整。确保玉米种植面积稳定在1000万亩，推动玉米产业发展。加快培育饲草产业，使之成为畜牧业发展的基本支撑。推进草原畜牧业转型升级，加快发展农区畜牧业，继续提高农区畜牧业在全市畜牧业中的比重和畜牧业在农牧业中的比重。

提高农牧业综合生产能力。推进“粮安工程”建设，粮食产量稳定在120亿斤以上。全市牲畜存栏突破2000万头只，肉类、牛奶产量稳定在25万吨和120万吨以上。加强农田草牧场基础设施建设，新增规模化养殖场100个、人工饲草地38万亩。新增节水灌溉48.5万亩，其中玉米膜下滴灌31万亩。农牧业机械化综合水平达到85%以上。

创新农村牧区经营制度。积极稳妥推进多种形式的适度规模经营，着力培育专业大户、家庭农牧场、农牧民专业合作社、农牧业企业等新型农牧业经营主体，健全企业与农牧民之间的利益联结机制。鼓励引导金融资本、工商资本和社会资本进入农牧业。推动土地、草牧场承包经营权流转、入股、信贷抵押等制度创新。

（四）大力发展现代服务业

全面推进旅游文化产业。明确旅游业为战略性支柱产业和结构调整的主攻方向，促进旅游与文化的深度融合。抓好旅游发展总体规划的实施，全面推进政府主导、企业主体、社会参与的旅游业发展模式。加快推进蒙古之源•蒙兀室韦民族文化园、呼伦贝尔两河圣山景区和旅游驿站等重点项目建设，启动5A级旅游景区创建工作，建设大兴安岭北部原始森林国家公园，打造国家级特色村寨。抓好以冰雪那达慕为品牌的冬季旅游，深度开发森林旅游和边境旅游。整顿和规范旅游市场秩序，提升服务水平。加强旅游宣传营销，全年旅游接待人数和旅游业总收入均保持两位数增长。

加快推进商贸物流业。重点推进呼伦贝尔经济技术开发区工业物流、海拉尔中俄蒙国际物流园区、大兴安国际物流园区、满洲里国际物流产业园区和滨洲铁路沿线城市物流带建设，推动物流产业尽快实现突破。加快万达、红星美凯龙、维多利等商业综合体的开工和建设。

继续促进金融业发展。完善金融服务体系，力争新设2家村镇银行。继续做大中小企业担保平台，扶持中小微企业发展。创新融资方式，推动更多企业直接融资。构建区域性产权交易市场，为中小微企业不动产抵押和农牧林业产权流转提供服务。探索排污权、收费权、土地承包经营权等质押贷款，推进土地、矿业权抵押融资。

加快培育新型业态。鼓励社会资金投入到养老机构、生活照料、医疗保健等健康养老产业。积极推动电子商务、快递配送、家政服务和环保产业等新型业态发展。

（五）统筹推进城乡一体化

加强城乡规划设计。高起点编制城镇特色景观风貌规划、美丽乡村规划、中心城区重点区域形象设计和地下管网规划。提升规划的科学性，加强对新型城镇化重大事项的研究，探索“三规合一”，积极引导和推动区域性中心城市、城关镇、重点镇协调发展，抓好扎兰屯国家级新型城镇化试点工作。强化规划的权威性，坚持一张蓝图绘到底。

提升城市综合承载能力。城市建设投资280亿元，新开工房屋1450万平方米。统筹抓好城市供热、燃气等360个市政基础设施项目。按照市区共建原则，加快中心城区提升改造步伐。结合居住小区既有建筑节能改造，加大老旧小区改造力度。提升物业服务水平，提高城市管理综合执法能力。完成市中心城区伊敏河、六二六小河河道整治及生态景观工程，启动中心城区供水扩容工程，继续实施“拆炉并网”工程，推动中心城区进入自治区园林城市行列。继续推动平安城市、智能交通系统和数字城市设施建设。

加快新城区建设。推进呼伦贝尔市中心城新区、海拉尔东山组团、满洲里、牙克石、扎兰屯等新城区建设，统筹抓好基础设施和公共服务配套建设。理顺呼伦贝尔市中心城新区规划执法和管理体制,加快中心城区一体化进程。

持续推进基础设施建设。公路投资90亿元，推进新林北—扎兰屯高速公路，海拉尔—伊敏、满洲里—阿拉坦额莫勒等一级公路建设，加快阿里河—库布春等一级公路前期工作。铁路投资27亿元，积极推进滨洲铁路电气化改造等项目建设，加快海拉尔—黑山头、室韦—莫尔道嘎等项目前期工作，争取齐齐哈尔—海拉尔—满洲里铁路客运专线项目列入国家“十三五”铁路网规划，并启动前期工作。民航投资5亿元，扎兰屯支线机场实现通航，继续抓好阿荣旗等5个通用机场建设，开工海拉尔机场扩建工程，加快中国民航大学内蒙古飞行学院建设。水利投资25亿元，推进扎敦水利枢纽和尼尔基水库下游30万亩灌区建设，开工Z866项目，加快毕拉河水利枢纽项目前期工作。电网投资30亿元，重点推进冯屯—岭东500千伏输变电、扎兰屯—阿荣旗—莫旗—大杨树220千伏输变电等工程。

推动区域协调发展。发挥比较优势，因地制宜，分类指导，加快县域经济发展。大力支持岭东地区加快发展绿色农畜产品加工业，推动岭上旅游、林下产品加工产业整合壮大和岭西煤电、现代煤化工、有色金属等产业转型发展。加强与森工、农垦及驻市中央企业和外埠大型企业的合作，形成发展合力。

（六）全面深化改革、扩大开放

按照中央和自治区2015年改革要点抓好各项改革工作。深化农村牧区改革。加快推进土地、草牧场确权登记工作，建立农村牧区土地草牧场流转交易平台。流转土地1000万亩以上、草牧场1500万亩以上。积极推进阿荣旗全国农村改革试验区工作。推动国有企业改革。组建国有资产监管和运营机构，规范国有资产监管工作。探索发展混合所有制经济，鼓励民间资本参与国有企业改革。继续培育壮大市场主体。推动农垦集团、城投集团等市场主体健全完善法人治理结构和现代企业制度，拓宽融资渠道。培育新的市场主体，发挥市场主体的投融资建设作用。深化商事制度改革。深入推进企业注册制度改革，最大限度实现便利化，释放市场活力。激发非公经济活力。按照“非禁即入”原则，努力营造良好投资环境和创业条件，切实解决非公经济融资难、贷款难等制约性问题。加强企业家队伍建设，推动非公企业提升实力、扩大规模。打造发展新优势。探索发展微电网，扩大大用户直供范围，建设企业、园区自备电厂，加快发展生产性物流业，着力解决大范围高效率配置资源问题。启动不动产统一登记。整合土地、房屋、草原、林地等不动产登记职责，搭建不动产统一登记平台。

紧紧抓住国家实施“一带一路”战略的历史性机遇，全力推动向北开放有新突破。争取国家支持，推动满洲里重点开发开放试验区政策放大适用范围。加快推进满洲里综合保税区获得国家批复，启动满洲里自贸区申报工作。统筹抓好“中俄”、“中欧”等货运班列运行。力争中蒙跨境经济合作区获得国家批复。启动合作区基础设施、跨境旅游、农畜产品加工、公共发电厂等先行性项目。加快推动呼伦贝尔中俄蒙合作先导区获国家批复。启动合作项目建设。推进与俄蒙的互联互通。加强口岸通关能力建设，积极推动满洲里至伊尔施沿边铁路建设，加快建设联通俄蒙、对接内地的国际大通道。争取额布都格和阿日哈沙特口岸常年开放。加大招商引资力度。创新思路、改进方法，组织精干小分队，按照产业结构、产业链条、产业集群的要求，开展针对性、专业性、实效性招商。突出对重点地区重点行业的招商，加强与东北、环渤海经济区、珠三角、长三角等地区的密切联系。

（七）持续保障和改善民生

继续加大公共财政对民生支出的倾斜力度，确保民生支出达到60%以上。

大力推进重点民生工程建设。着力推进“十个全覆盖”工程。投资22.1亿元，确保543个嘎查村实现全覆盖，再完成总任务量的45%，累计达到总量的80%。一定要做到任务、投资和质量三个确保，切实把这项全区最大的综合性民生工程抓紧抓实，惠及广大农牧民。切实抓好保障性安居工程。投资87亿元，开工建设各类保障性住房3万户，突出抓好林区棚户区续建和城市棚户区改造，抓住数量、质量、时限和分配等关键环节，确保完成任务。推进创业就业工程。加快推进各旗市区创业园孵化基地建设，以创业带动就业。认真解决困难群众特别是零就业家庭就业。城镇新增就业3.3万人，农牧民转移就业7.5万人，促进高校毕业生就业9000人。实施扶贫攻坚工程。继续开展金融扶贫、金种子扶贫工程，落实好“三到村三到户”项目，全面推进兴边富民行动，扶持三少民族和俄罗斯族发展，全年减贫35%以上。实施人才强市工程。拓宽人才引进“绿色通道”，抓好专业技术、高技能人才和农牧林区实用人才队伍建设。

加强社会保障。落实旗县以下机关公务员职务与职级并行制度、职工带薪休假制度和国家机关事业单位养老保险制度改革各项措施。严格执行社保提标扩面政策，确保社保基金运行安全。新农合财政补助标准由每人每年320元提高到380元。重点关注“三无”、低保和农村牧区五保等困难群体的生产生活。继续落实高龄老人津贴普惠制、低保家庭大学生入学资助和农村牧区低收入家庭冬季取暖用煤补贴政策。解决13.65万人的饮水安全问题。持续推进“菜篮子”工程，加强设施蔬菜基地建设，新增蔬菜仓储设施3.6万立方米、蔬菜平价店15个。

（八）加快发展社会事业

实施创新驱动战略，打造呼伦贝尔科技创业孵化基地，加快县域生产力促进中心机构建设。协同创新，实施农牧林业种子工程。推动各级各类教育全面协调发展，重点抓好义务教育基本均衡县创建工作，高度重视民族教育，加快完善现代职业教育体系，推进高等学校转型发展，实现扎兰屯职业学院秋季招生。加快现代公共文化服务体系建设，加大非物质文化遗产保护力度，推进蒙古族源和元朝帝陵综合研究项目，加快呼伦贝尔自然博物馆建设。完成自治区和市本级地面数字电视工程建设任务。开展县级公立医院改革，加强城乡一体化医疗卫生服务体系和卫生信息化建设，抓好重大疾病防控。促进人口长期均衡发展。加强食品药品监管体系和应急处置能力建设，完善应急预警体系，确保公共安全。抓好校园足球普及，推动全民健身和竞技体育同步发展。发展公益慈善事业，做好残疾人工作。深入开展“中国梦•尽责圆梦”系列主题实践活动，大力培育和践行社会主义核心价值观，提升市民道德素质和社会文明水平。做好“十三五”规划编制工作。统筹抓好统计、气象、人防、市场监管、地震和档案史志等工作。

（九）打造北疆生态安全屏障和安全稳定屏障

筑牢北疆生态安全屏障。全面贯彻新《环保法》，认真落实全市生态环保大会精神，把生态环境保护放在更加突出的地位，坚持源头严防、过程严管、后果严惩，标本兼治多管齐下。加快生态文明制度建设，划定生态红线，最大限度地增加生态资产，减少环境负债，为建设美丽中国提供生态保障。做好呼伦湖生态环境综合治理，出台《呼伦湖国家级自然保护区管理条例》，推动《呼伦湖生态保护与治理总体规划》获得国家批准。抓好呼伦湖周边生态恢复和部分封湖休渔，加强综合监测体系建设。全面落实国家主体功能区规划，实行最严格的源头保护制度。切实加强草原生态保护，落实好草原生态保护补助奖励等各项政策，退牧还草150万亩、禁牧2851万亩、草畜平衡7508万亩，继续治理沙区100万亩。抓好退耕还林、天保二期和重点公益林保护工程。继续深化国有林区改革，增强国有林区的生态功能和发展活力，争取将地方国有林区纳入国家停止商业性采伐范围，更多地享受国家政策支持。深入开展绿色矿山、和谐矿区建设，推进矿山地质环境综合整治。巩固森林城市创建成果，完成8万亩重点区域绿化和中心城区20公里环城绿化工作。深入开展水污染防治，实行最严格水资源管理制度，做好额尔古纳河、嫩江等重点流域环境综合整治，完成各旗市区和重点工业园区污水处理厂建设。探索编制自然资源资产负债表，加快建立生态环境损害责任终身追究制度，加大环保执法力度，对违法排污企业严管重罚。

推进北疆安全稳定屏障建设。认真贯彻执行党的民族政策，大力发展民族经济，繁荣发展民族文化，促进各民族共同团结进步，共同繁荣发展。坚持系统治理、依法治理、综合治理、源头治理，在法治轨道上统筹社会力量、平衡社会利益、调解社会关系、规范社会行为。提高社区治理服务水平，构建社区服务体系，依法推进嘎查村村民委员会换届工作。把信访纳入法治化轨道，健全依法维权和化解纠纷机制，实行重大决策社会稳定风险评估机制，继续开展领导干部下访接访，妥善解决群众合理诉求。深化“平安呼伦贝尔”建设，完善立体化社会治安体系，依法严厉打击各类违法犯罪活动，维护良好的社会治安环境，切实增强群众的安全感和满意度。加强安全生产，落实“一岗双责”，坚决遏制重特大安全事故。严格落实大型活动安保责任措施。高度重视网络舆情监管和舆情引导。强化信息化管边控边各项措施，进一步提升边境综合防卫管控能力。做好全国双拥模范城迎检工作，巩固良好的军政军民关系，维护民族团结、边疆安宁、和谐稳定的良好局面。

（十）加快法治政府建设

落实党的十八届四中全会精神，全面推进法治政府建设。

依法全面履行政府职能。用法治思维和方式履行职责，认真执行人大及其常委会各项决议、决定，依法向人大及其常委会报告工作，自觉接受政协民主监督。认真办理人大代表建议和政协委员提案，广泛听取各民主党派、工商联、无党派人士及各人民团体的意见和建议。继续推动简政放权。推进政府机构、职能、权限、程序、责任法定化，向社会公开政府权力清单、责任清单、负面清单，完成全市行政权力清理规范工作。

健全依法决策机制。认真执行市政府重大行政决策程序规定和合法性审查制度，建立和完善重大决策责任追究制度和倒查机制。完善法律顾问制度，组建政府法律顾问专家库，提高依法决策水平。严格规范公正文明执法。完善行政执法程序，落实行政执法主体责任。紧紧围绕关系群众切身利益、群众反映强烈的问题，加大执法力度。强化行政复议，进一步完善执法责任追究机制。推进“六五”普法工作，促进公正司法、全民守法。

全面推进政务公开。进一步加大政府信息公开力度，重点推进财政预决算、公共资源配置、社会公益事业建设的政府信息公开。面向社会服务的政府部门要全面推行办事公开制度，不断提高政府工作法制化水平和便民服务水平。

强化公共服务。所有审批项目必须全部进入政务服务中心，优化办事流程，简化办事手续，提高办结时效性。所有公共资源交易项目全部纳入公共资源交易中心集中交易，使市场对资源配置起决定性作用，努力营造公平的竞争环境，释放市场活力。积极推进政府购买服务工作。按规定推进全市公务用车改革。创新社会组织管理，让市场和社会组织承担更多事务。

坚持勤政廉政两手抓。贯彻落实“三严三实”要求，严格执行中央八项规定、国务院“约法三章”、自治区28项配套规定和市委有关要求，严控会议规模，精简文件数量，减少事务性活动，“三公”经费再压缩5%。认真落实党风廉政建设责任制，健全政府系统风险防控体系。加强行政监察、审计和财政监督。加大对工程招投标等重点领域和关键环节的监管。加强行政机关效能建设，严厉问责不作为、慢作为、乱作为等行为。巩固党的群众路线教育实践活动成果，推进作风建设常态化，扎实开展“万名干部下基层大引领行动”，多为群众办好事、解难题，打造为民、务实、清廉政府。

各位代表，今年经济社会发展的目标任务已经确定。让我们在以习近平为总书记的党中央领导下，守望相助，团结奋斗，扎实做好改革发展稳定各项工作，为把祖国北疆这道风景线打造得更加亮丽而努力奋斗！
